# Influence of Urban Green Space and Facility Accessibility on Exercise and Healthy Diet in Hong Kong

**DOI:** 10.3390/ijerph16091514

**Published:** 2019-04-29

**Authors:** John W. M. Yuen, Katherine K. P. Chang, Frances K. Y. Wong, Fiona Y. Wong, Judy Y. M. Siu, H. C. Ho, M. S. Wong, Janice Y. S. Ho, K. L. Chan, Lin Yang

**Affiliations:** 1 School of Nursing, The Hong Kong Polytechnic University, Hung Hom, Kowloon, Hong Kong, China; katherine.chang@polyu.edu.hk (K.K.P.C.); frances.wong@polyu.edu.hk (F.K.Y.W.); janice.ys.ho@polyu.edu.hk (J.Y.S.H.); kalong.chan@polyu.edu.hk (K.L.C.); l.yang@polyu.edu.hk (L.Y.); 2 School of Optometry, The Hong Kong Polytechnic University, Hung Hom, Kowloon, Hong Kong, China; fionawong1@gmail.com; 3 Department of Applied Social Sciences, The Hong Kong Polytechnic University, Hung Hom, Kowloon, Hong Kong, China; judy.ym.siu@polyu.edu.hk; 4 Department of Urban Planning and Design, The University of Hong Kong, Pok Fu Lam, Hong Kong, China; hcho21@hku.hk; 5 Department of Land Surveying and Geo-Informatics, The Hong Kong Polytechnic University, Hung Hom, Kowloon, Hong Kong, China; ls.charles@polyu.edu.hk

**Keywords:** physical activity, exercise, green space, open space, Metabolic Equivalent of Task, International Physical Activity Questionnaire, health promotion

## Abstract

*Background* A cross-sectional study using a convenience sampling method was conducted to understand how green space and accessibility of common public open spaces in compact urban areas affect physical activity and healthy diets of residents. *Methods* A total of 554 residents completed a structured questionnaire on quality of life, physical activity level and healthy eating practice. Particularly, categories of physical activity and durations were obtained by using the short form Chinese International Physical Activity Questionnaire (IPAQ-C), then the Metabolic Equivalent of Task (MET)-minutes/week was calculated using the formulae (walking minutes × walking days × 3.3) + (moderate-intensity activity minutes × moderate days × 4.0) + (vigorous-intensity activity minutes × vigorous-intensity days × 8.0). The percentage of green space was calculated based on a spatial buffer with a 500 m radius from participants’ geocoded addresses using a SPOT (‘Satellite Pour l’Observation de la Terre’ in French) satellite image-derived vegetation dataset. Parks, promenade and sports facilities were examples of open spaces. *Results* The sampled population who lived with green space averaged 10.11% ± 7.95% (ranged 1.56–32.90%), with the majority (90%) performing physical activities at medium and high levels. MET-minutes/week was significantly associated (Pearson *r* = 0.092; *p* < 0.05) with the green space percentage. Relatively active residents commonly used open spaces within the district for performing exercise, in particular, parks and promenades were mostly used by older residents, while sports facilities by the younger groups at age 25–44 and <25 years. *Conclusions* Current findings suggested promotion of exercise could be achieved by the design or redesign of built environment to include more parks accessible to the residents with the increase of vegetation.

## 1. Introduction 

Physical activity and healthy eating are the two important aspects of a healthy lifestyle [1,2,3]. A sedentary life together with excess energy intake particularly leads to the consequence of obesity, the major risk factor for mortality and many chronic problems including cardiovascular diseases [4,5], diabetes [6,7] and cancers [8,9]. The World Health Organization (WHO) recommended at least 150 min of aerobic physical activity at moderate-intensity, corresponding to 3–6 Metabolic Equivalents of Task (METs) per week for adults at age between 18 and 64 years, in order to improve cardiorespiratory and muscular fitness, bone health, reduce the risk of non-communicable diseases and depression [10]. The Hong Kong population was physically inactive in general, where three out of four Hong Kong residents had not participated a substantial level of physical activity with 36.1% being not active at all [11] and 59% living a sedentary lifestyle that did not involve any sports or exercise over a month [12]. In addition, more than half of the local population were reported to have no leisure time of physical activity over a 10-year period while 20.6% of all-cause deaths were attributable to not exercising [13]. Leisure-time physical activity was demonstrated to have protective effects able to reduce 37% and 25% of mortality in men and women, respectively [13]. In an international study involving 20 countries, the reported local prevalence of ‘low active’ (i.e., less than a total 600 MET-minutes per week) was contradictorily low at 15.3% for the population at age 20–64 years [14]. However, results of the recent Behavioral Risk Factor Survey 2016 indicated that almost half (43.8%) of Hong Kong’s adults had met the physical activity level recommended by the WHO while 18.5% and 21.1% of the studied population had a Body Mass Index (BMI) being classified as overweight and obese, respectively [15].

Besides physical activity, the same survey also reported the dietary patterns of the participants who consumed 3.4 servings of fruits and vegetables per day while three-quarters (73.7%) of them consumed more than 151 g (the recommended consumption threshold) of meat per day [15]. In addition to physical inactivity, the high prevalence of obesity in Hong Kong was believed to be associated with the typical unhealthy dietary pattern of many developed societies characterized by energy dense processed food typically high in fat, protein and refined carbohydrates with a low fiber content [16]. Obesity seemed to be promoted by the intake of variety of snacks while such weight gains could be reversed by the intake of a variety of grains and meats [17]. Additional contributing factors for obesity included sleeping and working hours [18] as well as night shift work [19] that also associated with general quality of life (QoL). People with a higher education level were prone to having a healthier diet that may lead to a lower prevalence of overweight individuals [20].

Accumulating evidence has suggested that there is a relationship between green space and health [21,22,23,24]. The systematic review of Lachowycz and Jones [21] identified inconsistent and mixed evidence on the positive impacts of green space toward obesity-related health indicators such as BMI, body fat and waist circumferences; likewise, the relationships between green space and physical activity were controversial. In the Netherlands, a study has demonstrated the correlations of green space with reduced occurrence of coronary heart disease and diabetes [25]. Empirical evidence also supported physical activity as the possible mechanism underlying the relationship between green space and health [22]. According to the Danish national representative survey, a better health-related quality of life (HRQoL) was observed when an individual was living closer to green spaces [26]. Green space can be defined by one of two interpretations: (1) refers to bodies of water or areas of vegetation in a landscape, which can be an antonym of urbanization; and (2) represents urban vegetation that is related to a vegetated variant of open space [27]. This study adopted the former definition, and green space was measured using a vegetation dataset derived from the SPOT (‘Satellite Pour l’Observation de la Terre’ in French) satellite images with the land use information in Hong Kong [28,29]. Urban green spaces, including parks and public recreation facilities, benefit the general public health through promoting physical activity and psychological well-being among urban residents [23]. In this study, open space was categorized as urban green space according to the Hong Kong Planning Standards and Guidelines [23,30].

Hong Kong is a compact city with most of its seven million population living in the urban areas at medium to high density. Although the whole territory of Hong Kong has a higher percentage of green space (51.2%) as compared with the nearby main cities in China, most of these are woodland and shrub land located at the countryside that are inaccessible to urban residents [31]. In fact, the green spaces in high-density areas of Hong Kong are totally fragmented while a small proportion of greens may be accessible to some medium-density areas (especially those newly developed areas on reclaimed land), whereas the overall open space-to-total space ratio is approximately 10% [32]. Our recent QoL study [33] demonstrated different levels of satisfaction with the physical environment and open spaces among the residents of nine districts of Hong Kong at medium-to-high density. Results of this study also indicated that around 60% of the studied population had sometimes or always participated in healthy eating with low fat, low sodium and low sugar, while 62.9% performed moderate physical activity regularly [33]. Individual adults are recommended to consume at least two portions of fruit and three portions of vegetable per day [34]. Therefore, we are particularly interested in further understanding how such healthy lifestyle practices are associated with the urban green space. In this study, a cross-sectional study was conducted to understand the relationships between green space and healthy lifestyle, particularly in physical activity levels and dietary habits in the nine urban residential areas of Hong Kong. Secondarily, the usage of recreational facilities, as examples of public open space accessible to the relatively physically active residents, and their characteristics, would also be explored.

## 2. Materials and Methods

### 2.1. Target Population and Recruitment

The studied population consisted of an existing dataset expanded by continuing the recruitment of residents from the nine district council constituency areas of Hong Kong that covered mixed-use commercial and residential districts, urban and more affluent districts with different housing types, and people with various socio-economic statuses [33]. Adult residents of any gender aged at least 20 years and had been living in any of the nine districts for more than one year were the target population. Those who were cognitively impaired, unable to communicate effectively in Cantonese, Mandarin or English, or having physical immobility that limits their physical activity were excluded. The convenience sampling method was used for recruiting the participants of this cross-sectional study. In brief, well-trained interviewers were allocated at parks, rest areas and outside food markets and shopping centers in the nine district areas from 9 a.m. to 7 p.m. on weekdays and weekends to capture all types of residents. Verbal consent was obtained from each participant after confirming the eligibility and explaining the purpose of the study. The answered questionnaire and signing for the token of appreciation were also implied consent to participate in this study. The whole procedure took around 15 min to complete. A supermarket shopping voucher (Hong Kong Dollar 50 value) was given to each of the participants at the end as an incentive. Ethical approval (Reference: HSEARS20170825001) was obtained from the Human Subjects Ethics Committee of the Hong Kong Polytechnic University.

### 2.2. The Instrument and Measurements

The structured instrument used in this study was composed of four major parts. First, the socio-demographic profiles of the residents were provided. Second, the validated 26-item “Hong Kong version of WHOQOL-BREF” questionnaire were used to assess the four domains of QoL perception, namely physical, psychological, social and environment with 24 items, in addition to two individual items on general health and overall QoL [35]. The domain scores were transformed into a linear scale between 0 and 100 following the scoring guideline. Third, the Chinese version of the International Physical Activity Questionnaire short form (IPAQ-C) was used for assessing the physical activity levels of the residents [36]. Participants were asked on the number of days in the past seven days prior to the survey and the daily time performing walking (as low-intensity), moderate and vigorous activities. Any kinds of walking were counted, including walking at work and at home, walking to travel from place to place, and any other walking that the participants did solely for recreation, sport, exercise or leisure. The physical activity levels were first categorized as low, medium and high, based on the scoring guidelines [37]. Then, the total MET-minutes/week was calculated using the formulae (walking minutes × walking days × 3.3) + (moderate-intensity activity minutes × moderate days × 4.0) + (vigorous-intensity activity minutes × vigorous-intensity days × 8.0). Lastly, the healthy eating practice (low sugar, low salt, low fat) was measured on a four-point Likert scale (1 = never; 4 = always), in addition to the fruit and vegetable intakes. Additionally, the usage of parks, promenades, outdoor and indoor sports facilities within and nearby the residential district of participants were also assessed, in terms of frequency and duration.

To measure the green space, the territory-wide green space data of a vegetation dataset derived from the SPOT satellite images was first converted into raster-based format with 10 m resolution [28,29]. Addresses of all participants were geocoded to the HK 1980 grid coordinates, and the focal statistics of ArcGIS 10.6 (Environmental Systems Research Institute, Redlands, CA, United States) was applied to calculate the percentage of green space within a 500 m radius buffer around each participant’s residence. The residential green space of the current studied population ranged from 1.47% to 33.89%, which was categorized at equal proportion into low (1.47%–11.94%), medium (11.95%–22.42%) and high (22.43%–32.89%) levels according to the green space % (Table 1).

### 2.3. Data Processing and Analysis

Data collected was analyzed using IBM SPSS Statistics 25.0 (Armonk, NY, USA). Individuals who failed to answer all items of the IPAQ-C were removed from the analysis, and two participants were excluded for this reason. Descriptive statistics (frequency and percentages) were used to describe the socio-demographics, the IPAQ levels and other continuous and categorical variables. Continuous variables and scores were summarized as mean and standard deviation (SD). A Chi-squared test was used to compare nominal variables, whereas the student’s *t*-test and one-way ANOVA tests were used for comparison of mean values between two groups and among multiple (>2) groups, respectively. Linear correlation between two variables was evaluated using the Pearson’s correlation analysis.

## 3. Results

### 3.1. Socio-Demographics and Living Environment of Participants

A total of 554 participants completed the questionnaire. The mean green space within a 500 m radius of participants’ residential addresses was 10.11% (SD = 7.95%), and many of the socio-demographic variables and WHOQoL scores were significantly different between the discrete subgroup levels (low, medium and high) of green space (Table 1). The majority of the studied population (61.0%) was living in an environment with low green space ranging between 1.56% and 9.88%, with the largest household size at 3.53 ± 1.64 people, but the highest monthly incomes with half graduated from college or above, the female-to-male ratio was about 2:1, and two-thirds were married and living at self-owned housing (Table 1). In contrast, only 14.6% of the studied population was living with high green space, was the oldest subgroup with 45% that had reached retirement age (≥65 years), with the lowest incomes, and lived the longest at the present address for 21.62 ± 13.65 years, with the majority living with family at household size of 3.30 ± 1.63 people (Table 1). The profile of the “high” green space subgroup matched with the majority (almost 70%) who were living in the rental type of housing, which was presumably public housing that was constructed by the government to provide considerable green space with outdoor space and facilities. Nonetheless, among the three subgroups, the “medium green space” was found to be the youngest and highest educated with moderate incomes, the smallest household size with almost half being single, and 14.07% living alone (Table 1). This “medium green space” subgroup seemed to be formed by approximately half-and-half rental and self-owned housing. Among the three subgroups, the “high” and “medium” green spaced participants perceived the best and poorest quality of life (QoL) in all four domains (Table 1).

### 3.2. Green Space Was Association with the Physical Activity Level of Participants

Physical activity of the studied population was measured in terms of MET-minutes/week and IPAQ levels. As shown in Table 2 and Table 3, the MET-minutes/week increased with the increased levels of green space (*p* < 0.031), although, the correlation coefficient was weak at 0.092 (*p* < 0.05). Regarding the IPAQ levels, the “medium” and “high” green space subgroups tended to perform moderate-to-high levels of physical activity while the physical activity levels of those living with low green space were mainly at moderate level (Table 2). Besides the green space, both the MET-minutes/week and IPAQ level were weakly correlated with the physical (*r* = 0.11–0.13; *p* < 0.01) and psychological (*r* = 0.10–0.12; *p* < 0.05) domains of the WHOQoL scale (Table 3). Demographically, weak correlations were only identified between the MET-minutes/week and education level (*r* = 0.092; *p* < 0.05) positively but monthly income (*r* = −0.102; *p* < 0.05) negatively (Table 3). However, together tested with the individual monthly income and educational level using the multinomial regression analysis, the green space level was shown to be a non-significant predictor for moderate and high IPAQ levels when compared with the low IPAQ level (Table 4).

On the other hand, participants living with both green space extremities were demonstrated to have similar dietary habits, which adopted, in general, the healthy style of low in fat, sodium and sugar consumption and a high vegetable and fruit content (Table 2). However, the dietary habits of the “medium green space” subgroup were relatively unhealthy with the majority eating <1 serving of vegetables (58%), <1 serving of fruits (40%), and at least one-quarter (25.93%–30.37%) of them seldom adopted low fat, low sodium and low sugar diets (Table 2). Unlike physical activity, the green space of the participants’ residency was not correlated with the healthy dietary habits, except for the high vegetable consumption that was negatively significantly correlated (*r* = quality of life 0.087; *p* = 0.041) (Table 3). However, many of the dietary habit components were correlated with different demographic and WHOQoL variables (Table 3).

### 3.3. Accessibility of Outdoor Open Space Facilities in the Residential District Promotes Exercise

To understand how the major open spaces (parks and promenade) and sports facilities (outdoor and indoor) within and at nearby districts were used by the residents who had performed significant levels of physical activities, only participants with high and moderate IPAQ levels (representing 90% of the entire population studied) remained for further analyses. The MET-minutes per week values significantly (*p* < 0.001) varied among different age groups of the active participants, with the highest at age 45–64, followed by age ≥65 then age <25 and 25–44 (Table 5). Districts with parks were the most frequent open space facility being used by up to 54% of active daily users, whereas both the frequency and duration of usage increased linearly with age (Table 5). Up to 35% of younger residents used the promenade within the district on a weekly basis, but most of the daily users were those at older age with 7.5% at age 45–64 and 14.4% at age ≥65, while the duration of usage also followed the linear increasing trend with age (Table 5). Regarding the sports facilities, irrespective of indoor or outdoor, they were prompted to be used more frequently at longer duration by the younger age groups (Table 5). On the contrary, only around 10% of all ages of active participants travelled at a 2–4 times per week frequency to the facilities of nearby districts for spending less an hour per month on average, although significant variations (*p* < 0.01) were observed among different age groups (Table 4). However, the participants at high IPAQ level were living with a significantly higher (*p* < 0.01) residential green space than those of moderate level (Table 6). Those higher physical activity residents were shown to use all facilities within district as well as at nearby districts more frequently and for a longer duration than the moderately active counterparts (Table 6). Current results suggested that, irrespective of age (Table 5) and physical activity level (Table 6) of participants, active residents used predominantly the facilities within their districts, whereas the green space was also shown to be a promoting factor for performing exercises.

## 4. Discussion

The studied population was formed by residents of typical urban areas that are covered by a limited range of vegetation. The physical activity of participants as measured by MET-minutes per week and IPAQ levels were positively related with the green space percentage. The majority of participants had performed regular exercise at moderate and high levels. Irrespective of age and physical activity level, those active participants used predominantly the facilities within their residential districts, but facilities at nearby districts were seldom used. Particularly, parks and promenades were mostly used by older residents while sports facilities by the younger groups. Results suggested open space facility accessibility was an important promoting factor for exercises in compacted urban areas, in addition to the level of green space. On the other hand, healthy eating habits were not correlated with the green space but other demographic and QoL variables.

The studied districts areas represented the living environment with the highest population density and lowest green space, where the green spaces were fragmented and embedded in the built-up areas [32]. The current results also indicated the studied districts represented a typical lower-to-middle socio-economic class population of Hong Kong, whereas the majority were found to be physically active, meeting the WHO’s recommendation to perform at least 150 min of moderate-intensity exercise in a week [10]. With around 60% and 10% of participants being categorized, respectively, as having moderate and low physical activity levels, the pattern of this extended study population was consistent with that reported in our previous publication [33]. These findings also agreed with the results of an international study involving 14 urban cities, where Hong Kong was identified as one of the upper bound range cities with 56% of adult residents meeting the 150 min/week guideline who participated on average 44.9 min each day on moderate-to-vigorous physical activity [38]. These findings were contradictory to the notion of high physical inactivity of Hong Kong that only one-third of the population had met the WHO guidelines [11,12]. The adequate physical activity knowledge among the general population of Hong Kong may explain the increase of physical activity practice in the past two decades [39], which required further elucidation. Current results were also inconsistent with many studies that reported a negative correlation between physical activity participation and socio-economic status among urban living participants [40,41,42]. In Hong Kong, the government provides public rental housing estates to the low socio-economic population at an affordable cost. Those public housing estates are built with greener and healthier designs to provide a considerable recreational spaces for different activities [43], which was in contrast with the private housing where all the shared spaces and facilities are paid for by the owners [44]. Therefore, within the compact urban areas, residents of lower socio-economic are common living in housing with relatively higher green spaces and more shared spaces than the high socio-economic counterparts. Besides, it was suggested that higher education level in the Hong Kong Chinese population was associated with a healthier diet that leads to lower prevalence of obesity and certain cardiovascular risks [20]. Despite this, there were statistically significant correlations in this study, where higher education levels of participants were associated with poorer dietary habits with lesser consumption of low sugar, low salt, low fat, and high fiber diets. Since the pattern of dietary habits were not correlated with the urban green space, its inter-relationship with other demographic and QoL factors will be further studied.

It is well established that urban green spaces have multiple health benefits, and lower socio-economic groups such as elderly, youth and those less educated were seemed to benefit more from the green areas of their living urban environment [24,45]. The strongest health benefit of green space has been related to obesity [21]. The positive relationship between green space % and physical activity identified in this study supported the notion of physical activity as a possible mechanism for the health benefits derived from green spaces [22]. Urban green spaces at neighborhood areas were frequently visited by over 70% of Hong Kong residents, whereas physical exercise and strolling ranked as the top purpose [46]. Besides green spaces, numerous studies suggested accessibility to public open spaces was a key environmental determinant affecting physical activity participation [42,47]. Participations of physical activity among adults in 11 countries were found to be associated with the accessibility of certain built environment characteristics at the neighborhood with the highest odds for sidewalks present [48]. At the community level, public spaces and sports facilities serve multiple functions leading the behavioral choices of different physical activities [46]. Without close access to fitness facilities was considered as one of the significant barriers for performing physical activity [49]. The current study identified parks within district as the main public open spaces used by the relatively physically active residents. This was consistent with the positive association between the number of parks and participation in physical activity at moderate and vigorous levels across 14 urban cities including Hong Kong [38]. In addition to accessibility, several other attributes including cleanliness, aesthetically appealing, and safeness of parks were perceived by users for encouraging use across the life-span [50]. Owing to the limitation of cross-sectional design, path analysis will be performed as future study for determining the causal relationship between exercise, green space and facility accessibility. Furthermore, the present study was also limited to the convenience sampling method, as well as the green space only being measured in a 500 m radius surrounding each participant’s residence.

## 5. Conclusions

Green space and accessibility of public open spaces were positively associated with the physical activeness of residents living in old compact urban areas of Hong Kong. However, no association was observed between the dietary habits and green space. This suggests promotion of exercise can be achieved by the design or redesign of built environment to include more parks that are accessible to residents with the increase of vegetation.

## Figures and Tables

**Table 1:** Socio-demographic characteristics and quality of life (QoL) scores of participants living with different green space levels.

Variables		Total	Green Space Levels			
			Low	Medium	High	χ^2^ Test or One-Way ANOVA
		*n* = 554	*n* = 338	*n* = 135	*n* = 81	
		Frequency (Percentage)				
**Green space (%)**	Mean ± SD, range	10.11 ± 7.95, 1.56–32.90	4.38 ± 1.90, 1.56–9.88	16.12 ± 3.25, 10.16–19.62	24.05 ± 2.69, 20.02–32.90	F = 2722.80; *p* <0.001
**Age (years old)**	<25	101 (18.23)	47 (13.91)	47 (34.81)	7 (8.64)	χ^2^ = 46.17; *p* = 0.001
	25–44	165 (29.78)	105 (31.07)	39 (28.89)	21 (25.93)	
	45–64	132 (23.83)	90 (26.63)	26 (19.26)	16 (19.75)	
	≥65	156 (28.16)	96 (28.40)	23 (17.04)	37 (45.68)	
	Mean ± SD	48.05 ± 20.98	49.53 ± 20.45	39.90 ± 20.14	55.47 ± 20.60	F = 17.02; *p* < 0.001
**Gender**	Male	198 (35.74)	117 (34.62)	52 (38.52)	29 (35.80)	χ^2^ = 0.64; *p* = 0.726
	Female	356 (64.26)	221 (65.38)	83 (61.48)	52 (64.20)	
**Marital status**	Single	168 (30.32)	87 (25.74)	64 (47.41)	17 (20.99)	χ^2^ = 28.72; *p* = 0.001
	Married	339 (61.19)	223 (65.98)	63 (46.67)	53 (65.43)	
	Divorced/widowed	47 (8.48)	28 (8.28)	8 (5.93)	11 (13.58)	
**Years been living in current district**		15.19 ± 14.09	15.31 ± 13.95	11.27 ± 13.32	21.62 ± 13.65	F = 14.32; *p* < 0.001
**Housing type**	Self-owned	303 (54.69)	218 (64.50)	60 (44.44)	25 (30.86)	χ^2^ = 38.42; *p* < 0.001
	Rental	251 (45.31)	120 (35.50)	75 (55.56)	56 (69.14)	
**Living status**	Alone	58 (10.47)	33 (9.76)	19 (14.07)	6 (7.41)	χ^2^ = 65.85;p < 0.001
	With someone	496 (89.53)	305 (90.24)	116 (85.93)	75 (92.59)	
**Household size**	Mean ± SD	3.21 ± 1.61	3.53 ± 1.64	2.77 ± 1.44	3.30 ± 1.63	F = 7.369; *p* = 0.001
**Educational level**	≤Primary	109 (19.68)	67 (19.82)	13 (9.63)	29 (35.80)	χ^2^ = 31.80; *p* < 0.001
	Secondary	166 (29.96)	101 (29.88)	38 (28.15)	27 (33.33)	
	≥College	278 (50.18)	170 (50.30)	83 (61.48)	25 (30.86)	
**Monthly income (HKD)**	No income	247 (44.58)	146 (43.20)	58 (42.96)	43 (53.09)	χ^2^ = 18.11; *p* = 0.020
	≤10,500	126 (22.74)	69 (20.41)	36 (26.67)	21 (25.93)	
	10,501–14,800	48 (8.66)	34 (10.06)	10 (7.41)	4 (4.94)	
	14,801–23,000	48 (8.66)	24 (7.10)	17 (12.59)	7 (8.64)	
	≥23,001	85 (15.34)	65 (19.23)	14 (10.37)	6 (7.41)	
**WHOQoL scores**	Physical	60.89 ± 1032	60.67 ± 10.38	59.45 ± 9.97	64.20 ± 10.54	F = 5.64; *p* = 0.004
	Psychological	62.92 ± 13.56	63.41 ± 13.23	60.68 ± 14.33	64.61 ± 13.28	F = 2.72; *p* = 0.067
	Social	62.69 ± 12.52	63.16 ± 12.41	61.03 ± 13.97	63.54 ± 10.05	F = 1.61; *p* = 0.200
	Environmental	62.15 ± 13.57	62.91 ± 12.21	58.92 ± 14.55	64.56 ± 12.51	F = 5.97; *p* = 0.003

HKD = Hong Kong Dollars; WHOQoL = The World Health Organization Quality of Life.

**Table 2:** Physical activities and dietary habits of participants living with different green space levels.

Variables		Total	Green Space Levels			Χ^2^ Test or
			Low	Medium	High	One-Way ANOVA
		*n* = 554	*n* = 338	*n* = 135	*n* = 81	
Physical Activities		Mean ± SD				
MET-minutes/week	Total	2421.80 ± 1785.51	2285.70 ± 1649.69	2505.50 ± 1874.95	2850.25 ± 2105.61	F = 3.49; *p* = 0.031
		**Frequency (Percentage)**				
IPAQ levels	High	179 (32.31)	94 (27.81)	53 (39.26)	32 (39.51)	χ^2^ = 8.90; *p* = 0.064
	Moderate	319 (57.58)	210 (62.13)	67 (49.63)	42 (51.85)	
	Low	56 (10.11)	34 (10.06)	15 (11.11)	7 (8.64)	
**Dietary habits**						
Fulfillment of 2 servings of fruits + 3 servings of vegetables		32 (5.78)	24 (7.10)	5 (3.70)	3 (3.70)	χ^2^ = 2.79; *p* = 0.247
Fruit consumption (serving per day)	≥2 servings	90 (16.25)	59 (17.46)	20 (14.8)	11 (13.58)	χ^2^ = 15.16; *p* = 0.019
	1 serving	212 (38.27)	135 (39.94)	37 (27.41)	40 (49.38)	
	<1 serving	231 (41.70)	133 (39.35)	72 (53.33)	26 (32.10)	
	None	21 (3.79)	11 (3.25)	6 (4.44)	4 (4.94)	
Vegetable consumption (serving per day)	≥3 serving	54 (9.75)	37 (10.95)	9 (6.67)	8 (9.88)	χ^2^ = 11.82; *p* = 0.066
	1–2 serving	340 (61.37)	215 (63.61)	72 (53.33)	53 (65.43)	
	<1 serving	158 (28.52)	85 (25.15)	53 (39.26)	20 (24.69)	
	None	2 (0.36)	1 (0.30)	1 (0.74)	0 (0.00)	
Low fat consumption	Often	166 (29.96)	103 (30.47)	33 (24.44)	30 (37.04)	χ^2^ = 15.48; *p* = 0.017
	Sometimes	270 (48.74)	176 (52.07)	67 (49.63)	27 (33.33)	
	Seldom	118 (21.30)	59 (17.46)	35 (25.93)	24 (29.63)	
Low sodium consumption	Often	169 (30.51)	105 (31.07)	33 (24.44)	31 (38.27)	χ^2^ = 13.43; p = 0.037
	Sometimes	263 (47.47)	170 (50.30)	65 (48.15)	28 (34.57)	
	Seldom	122 (22.02)	63 (18.64)	37 (27.41)	22 (27.16)	
Low sugar consumption	Often	186 (33.57)	119 (35.21)	35 (25.93)	32 (39.51)	χ^2^ = 18.77; *p* = 0.005
	Sometimes	247 (44.58)	28 (8.28)	59 (43.70)	6 (7.41)	
	Seldom	121 (21.84)	59 (17.46)	41 (30.37)	21 (25.93)	

IPAQ = International Physical Activity Questionnaires.

**Table 3:** Correlational analysis among variables measured in the studied population.

Variables		Dietary Habits					Physical Activity		WHO-QoL Scores				Demographics			
		Low Sugar	Low Salt	Low Fat	High Veg	High Fruit	IPAQ Level	MET-Min	Envir	Social	Psy	Phy	MI	Edu Level	HH Size	Age
		Pearson correlation coefficient (r); *p*-value														
Green space %		−0.083; 0.051	−0.077; 0.071	−0.074; 0.084	−0.087; 0.041	−0.077; 0.069	0.077; 0.071	0.092; 0.030	−0.023; 0.588	−0.056; 0.187	−0.029; 0.496	0.055; 0.199	−0.131; 0.002	−0.051; 0.227	−0.047; 0.268	−0.042; 0.325
Demographics	Age	0.229; <0.001	0.288; <0.001	0.252; <0.001	0.228; <0.001	0.169; <0.001	0.049; 0.247	0.059; 0.165	0.026; 0.537	−0.052; 0.223	−0.021; 0.630	0.046; 0.283	−0.308; <0.001	−0.722; <0.001	−0.044; 0.296	
	HH size	0.073;0.086	0.066; 0.122	0.040; 0.342	0.155; <0.001	0.110; 0.009	0.065; 0.124	0.082;0.055	0.028; 0.505	0.105; 0.013	0.153; <0.001	0.072; 0.091	−0.069; 0.103	0.000; 1.000		
	Edu level	−0.154; <0.001	−0.186; <0.001	−0.143; 0.001	−0.160; <0.001	−0.136; 0.001	−0.072; 0.089	−0.092; 0.031	0.094; 0.027	0.063; 0.138	0.090; 0.033	−0.033; 0.444	0.403; <0.001			
	MI	−0.035; 0.416	−0.073; 0.086	−0.072; 0.090	−0.139; 0.001	−0.045; 0.290	−0.071; 0.094	−0.102; 0.017	0.016; 0.716	0.038; 0.369	0.048; 0.256	−0.026; 0.540				
WHO-QoL scores	Phy	0.108; 0.011	0.088; 0.038	0.050; 0.243	0.198; <0.001	0.162; <0.001	0.133; 0.002	0.111; 0.009	0.503; <0.001	0.450; <0.001	0.627; <0.001					
	Psy	0.073; 0.087	0.047; 0.266	0.029; 0.489	0.187; <0.001	0.214; <0.001	0.097; 0.023	0.124; 0.003	0.608; <0.001	0.506; <0.001						
	Social	0.095; 0.026	0.105; 0.013	0.066; 0.120	0.175; <0.001	0.112; 0.008	0.039; 0.361	0.024; 0.573	0.436; <0.001							
	Envir	0.128; 0.003	0.077; 0.070	0.060; 0.156	0.111; 0.009	0.182; <0.001	0.044; 0.303	0.039; 0.363								
Physical activity	MET−min	0.002; 0.970	0.009; 0.831	−0.007; 0.877	0.213; <0.001	0.167; <0.001	0.806; <0.001									
	IPAQ level	−0.002; 0.966	0.001; 0.980	−0.015; 0.727	0.245; <0.001											
Dietary habits	High fruit	0.203; <0.001	0.251; <0.001	0.243; <0.001	0.560; <0.001											
	High veg	0.271; <0.001	0.299; <0.001	0.294; <0.001												
	Low fat	0.836; <0.001	0.893; <0.001													
	Low salt	0.846; <0.001														

Footnotes: MI = Monthly Incomes; HH = Household size.

**Table 4:** Results of multinomial regression analysis using the IPAQ levels as the dependent variable for testing the predictive values of educational levels, individual monthly incomes and green space levels.

Multinomial (χ^2^ = 41.14; *p* < 0.001)		Comparisons					
		Low Versus Moderate IPAQ Levels			Low Versus High IPAQ Levels		
		β_1_	SE (β_1_)	OR_1_	β_2_	SE (β_2_)	OR_2_
**Education**	**(Primary)**	0.536	0.484	1.709 (*p* = 0.269)	0.669	0.507	1.952 (*p* = 0.187)
	**(Secondary)**	0.196	0.384	1.216 (*p* = 0.610)	0.447	0.403	1.564 (*p* = 0.267)
	**(Diploma)**	−1.418	0.481	0.242 (*p* = 0.003)	−0.982	0.517	0.375 (*p* = 0.058)
	**(University)**	Reference			Reference		
**Income (HKD)**	**(0)**	−0.268	0.481	1.964 (*p* = 0.578)	0.287	0.522	1.333 (*p* = 0.582)
	**(≤10,500)**	−0.600	0.489	1.431 (*p* = 0.200)	−0.259	0.537	0.772 (*p* = 0.629)
	**(10,501–14,800)**	−1.037	0.554	1.050 (*p* = 0.061)	−0.668	0.615	0.513 (*p* = 0.278)
	**(14,801–23,000)**	1.598	1.097	42.447 (*p* = 0.145)	2.079	1.121	8.000 (*p* = 0.064)
	**(≥23,001)**	Reference			Reference		
**Green level**	**(Low)**	0.142	0.466	2.875 (*p* = 0.760)	−0.308	0.481	0.735 (*p* = 0.523)
	**(Medium)**	−0.182	0.521	2.315 (*p* = 0.726)	−0.072	0.534	0.930 (*p* = 0.893)
	**(High)**	Reference			Reference		

HKD = Hong Kong Dollars; OR = Odds Ratio.

**Table 5:** The usage of within and nearby district facilities by active participants of different age ranges.

Variables		Age Group				χ^2^ Test or One-Way ANOVA
		<25	25–44	45–64	≥65	
		*n* = 86	*n* = 146	*n* = 120	*n* = 146	
**MET-Minutes Per Week**	Mean ± SD	4326 ± 1398	4031 ± 1188	5118 ± 1631	4728 ± 1245	F = 5.799; *p* = 0.001
		Number (Percentage)				
**Usage of facilities—Within district**						
Parks	<Once a month	60 (69.8)	57 (39.0)	41 (34.2)	16 (11.0)	χ^2^ = 171.78; *p* < 0.001
	2–4 per month	19 (22.1)	33 (22.6)	31 (25.8)	11 (7.5)	
	>Once per week	7 (23.3)	34 (23.3)	25 (20.8)	40 (27.4)	
	Daily	0 (0.0)	22 (15.1)	23 (19.2)	79 (54.1)	
Hours used per month, mean ± SD		1.91 ± 4.90	13.64 ± 30.53	17.22 ± 34.42	41.14 ± 42.83	F = 30.28; *p* < 0.001
Promenade	<Once a month	48 (55.8)	80 (54.8)	66 (55.0)	90 (61.6)	χ^2^ = 44.37; *p* < 0.001
	2–4 per month	30 (34.9)	47 (32.2)	30 (25.0)	21 (14.4)	
	>Once per week	7 (8.1)	18 (12.3)	15 (12.5)	14 (9.6)	
	Daily	1 (1.2)	1 (0.7)	9 (7.5)	21 (14.4)	
Hours used per month, mean ± SD		2.33 ± 3.70	3.28 ± 7.71	6.50 ± 21.22	8.86 ± 19.45	F = 4.669; *p* = 0.003
Outdoor sports facilities	<Once a month	71 (82.6)	109 (74.7)	102 (85.0)	126 (86.3)	χ^2^ = 34.10; *p* < 0.001
	2–4 per month	12 (13.9)	23 (15.8)	14 (11.7)	6 (4.1)	
	>Once per week	3 (3.5)	11 (9.6)	4 (3.3)	4 (2.7)	
	Daily	0 (0.0)	3 (1.4)	0 (0.0)	10 (6.9)	
Hours used per month, mean ± SD		1.67 ± 5.07	3.72 ± 18.24	1.04 ± 3.29	2.44 ± 8.55	F = 1.382; *p* = 0.247
Indoor sports facilities	<Once a month	53 (63.0)	93 (63.7)	98 (81.7)	125 (85.6)	χ^2^ = 46.71; *p* < 0.001
	2–4 per month	22 (26.2)	37 (25.3)	16 (13.3)	7 (4.8)	
	>Once per week	11 (12.8)	14 (9.6)	5 (4.2)	9 (6.2)	
	Daily	0 (0.0)	2 (1.4)	1 (0.8)	5 (3.4)	
Hours used per month, mean ± SD		3.43 ± 7.12	2.88 ± 6.36	1.86 ± 8.16	2.63 ± 11.8	F = 0.576; *p* = 0.631
**Usage of facilities—Nearby districts**						
Parks	<Once a month	77 (89.5)	107 (73.3)	108 (90.0)	129 (88.3)	χ^2^ = 35.13; *p* < 0.001
	2–4 per month	8 (9.3)	37 (25.3)	11 (9.2)	10 (6.9)	
	>Once per week	1 (1.2)	2 (1.4)	0 (0.0)	4 (2.7)	
	Daily	0 (0.0)	0 (0.0)	1 (0.8)	3 (2.1)	
Hours used per month, mean ± SD		0.41 ± 1.07	1.64 ± 4.64	0.86 ± 6.85	1.80 ± 6.96	F = 1.508; *p* = 0.212
Promenade	<Once a month	76 (88.4)	126 (86.3)	110 (91.7)	143 (97.9)	χ^2^ = 21.01; *p* = 0.010
	2–4 per month	10 (11.6)	20 (13.7)	8 (6.7)	2 (1.4)	
	>Once per week	0 (0.0)	0 (0.0)	1 (0.8)	0 (0.0)	
	Daily	0 (0.0)	0 (0.0)	1 (0.8)	1 (0.7)	
Hours used per month, mean ± SD		0.34 ± 0.81	0.65 ± 1.68	0.77 ± 5.54	0.29 ± 2.51	F = 0.673; *p* = 0.569
Outdoor sports facilities	<Once a month	73 (84.9)	123 (84.2)	109 (90.8)	138 (94.5)	χ^2^ = 22.76; *p* = 0.009
	2–4 per month	11 (12.8)	21 (14.4)	10 (8.4)	4 (2.7)	
	>Once per week	2 (2.3)	2 (1.4)	1 (0.8)	1 (0.7)	
	Daily	0 (0.0)	0 (0.0)	0 (0.0)	3 (2.1)	
Hours used per month, mean ± SD		1.53 ± 5.05	1.83 ± 6.30	0.74 ± 2.87	1.12 ± 6.26	F = 0.999; *p* = 0.393
Indoor sports facilities	<Once a month	76 (88.4)	131 (89.7)	111 (92.5)	145 (99.3)	χ^2^ = 15.73; *p* = 0.020
	2–4 per month	8 (9.3)	12 (8.2)	6 (5.0)	1 (0.7)	
	>Once per week	2 (2.3)	3 (2.1)	3 (2.5)	0 (0.0)	
	Daily	0 (0.0)	0 (0.0)	0 (0.0)	0 (0.0)	
Hours used per month, mean ± SD		0.64 ± 1.93	0.59 ± 2.27	0.69 ± 2.90	0.10 ± 0.09	F = 3.258; *p* = 0.021

**Table 6:** Comparison of the usage of within and nearby district facilities among moderate and high IPAQ level residents.

Variables.		IPAQ Levels		χ^2^ Test ^†^ or Student’s *t*-Test
		Moderate (*n* = 319)	High (*n* = 179)	
		Mean ± SD		
**Green space %**		9.38 ± 7.69	11.35 ± 8.41	*p* = 0.008
**Age**		49.08 ± 21.01	48.12 ± 20.83	*p* = 0.626
**Years been living in current district**		15.18 ± 13.70	15.31 ± 14.78	*p* = 0.919
**HH size**		3.13 ± 1.65	3.37 ± 1.67	*p* = 0.112
		**Number (Percentage)**		
**Frequency of Facilities usage within district**				
**Parks**	<Once a month	119 (37.3)	55 (30.7)	*p* = 0.053 ^†^
	2–4 per month	66 (20.7)	28 (15.6)	
	>Once per week	66 (20.7)	40 (22.3)	
	Daily	68 (21.3)	56 (31.3)	
	Hours used per month, mean ± SD	18.11 ± 34.31	24.86 ± 38.56	*p* = 0.045
**Promenade**	<Once a month	186 (58.3)	98 (54.7)	*p* = 0.835 ^†^
	2–4 per month	81 (25.4)	47 (26.3)	
	>Once per week	32 (10.0)	22 (12.3)	
	Daily	20 (6.3)	12 (6.7)	
	Hours used per month, mean ± SD	4.76 ± 11.11	6.89 ± 21.44	*p* = 0.146
**Outdoor sports facilities**	<Once a month	274 (85.9)	134 (74.9)	*p* = 0.020 ^†^
	2–4 per month	29 (9.1)	26 (14.5)	
	>Once per week	10 (3.1)	12 (6.7)	
	Daily	6 (1.9)	7 (3.9)	
	Hours used per month, mean ± SD	1.60 ± 6.07	3.66 ± 16.87	*p* = 0.050
**Indoor sports facilities**	<Once a month	246 (77.1)	123 (68.7)	*p* = 0.044 ^†^
	2–4 per month	47 (14.7)	35 (19.6)	
	>Once per week	24 (7.5)	15 (8.4)	
	Daily	2 (0.6)	6 (3.4)	
	Hours used per month, mean ± SD	1.89 ± 4.02	4.02 ± 12.76	*p* = 0.010
**Frequency of Facilities usage in nearby districts**				
**Parks**	<Once a month	277 (86.8)	144 (80.4)	*p* = 0.113 ^†^
	2–4 per month	36 (11.3)	30 (16.8)	
	>Once per week	5 (1.6)	2 (1.1)	
	Daily	1 (0.3)	3 (1.7)	
	Hours used per month, mean ± SD	0.97 ± 4.32	1.85 ± 7.48	*p* = 0.096
**Promenade**	<Once a month	300 (94.0)	155 (86.6)	*p* = 0.013 ^†^
	2–4 per month	19 (6.0)	21 (11.7)	
	>Once per week	0 (0.0)	1 (0.6)	
	Daily	0 (0.0)	2 (1.1)	
	Hours used per month, mean ± SD	0.24 ± 0.96	1.03 ± 5.13	*p* = 0.008
**Outdoor sports facilities**	<Once a month	296 (92.8)	147 (82.1)	*p* = 0.001 ^†^
	2–4 per month	21 (6.6)	25 (14.0)	
	>Once per week	2 (0.6)	4 (2.2)	
	Daily	0 (0.0)	3 (1.7)	
	Hours used per month, mean ± SD	0.73 ± 2.34	2.34 ± 7.67	*p* = 0.001
**Indoor sports facilities**	<Once a month	303 (95.0)	160 (89.4)	*p* = 0.055 ^†^
	2–4 per month	13 (4.1)	14 (7.8)	
	>Once per week	3 (0.9)	5 (2.8)	
	Daily	0 (0.0)	0 (0.0)	
	Hours used per month, mean ± SD	0.33 ± 0.67	0.67 ± 2.56	*p* = 0.080

^†^ Determined by the χ^2^ test
